# The Evolution of Prostate Cancer Therapy: Targeting the Androgen Receptor

**DOI:** 10.3389/fonc.2014.00295

**Published:** 2014-10-24

**Authors:** Jeanny B. Aragon-Ching

**Affiliations:** ^1^Division of Hematology and Oncology, Department of Medicine, George Washington University Medical Center, Washington, DC, USA

**Keywords:** prostate cancer, androgen receptor, castration-resistant prostate cancer, abiraterone acetate, enzalutamide

Prostate cancer treatment has evolved through the varying eras of therapy from one of predominantly hormonal agents without much effective cytotoxic therapy until the use of docetaxel was found to improve overall survival in the pivotal TAX-327 (1) and SWOG 99-16 trials (2). While efforts to search for the appropriate docetaxel partner has yielded disappointing results with multiple Phase III trials showing negative results, further hormonal manipulation steadily gained ground upon discovery of persistent hormonal signaling with the use of novel androgen-biosynthesis inhibitors and anti-androgens. However, resistance to these agents is ultimately inevitable. Increased understanding of these resistance mechanisms may help re-channeling efforts toward better refinement and improvement of drug therapies.

Prostate cancer remains the second leading cause of death in the United States. While treatment for early-stage low-risk prostate cancer has been largely controversial with the advent of the prostate specific antigen (PSA) screening controversy, treatment for metastatic castration-resistant prostate cancer (mCRPC) has evolved with great strides in the past decade, though remains incurable to this day. Docetaxel as a treatment for mCRPC brought about improvement in overall survival (1). To date, no appropriate docetaxel partner has been found to be beneficial. However, the utility of androgen targeted signaling quickly gained ground with the discovery of the relevance of androgen receptor (AR) targeting long after failure from androgen deprivation therapy (3). This brought about the discovery and subsequent approval of both abiraterone acetate (4, 5) and enzalutamide (6, 7) in both the post-docetaxel and pre-docetaxel space. While targeting androgen-signaling to date makes for one of the most attractive approaches in mCRPC therapy, several challenges remain.

The AR is a 110-kDa steroid receptor encoded by the gene located in Xq11-12 in the same family of nuclear hormone receptors as the estrogen, progesterone, and glucocorticoid receptors (8). The AR functions as a “lineage oncogene” of which prostate tumors become habitually addicted to (9). Conversely, several AR-pathway genes are down-regulated upon progression from a low-grade to high-grade prostate cancer or in the development of metastases (10). Despite targeting the AR pathway using more contemporary drugs with abiraterone or enzalutamide, it is now increasingly recognized that resistance patterns are born at the cellular level, with ligand independent AR activation, AR mutation, intratumoral androgen synthesis, increased AR mRNA expression, and AR maintenance by heat shock proteins and AR structural alterations including acquisition of splice variants all playing a role in resistance (11). Given the inevitable notion of resistance, efforts toward not just sequencing, but combining these agents offer exciting insights and opportunities for treatment. For instance, TOK-001, also known as galeterone, is a rationally designed compound that has triple mechanism of action that includes inhibition of CYP17A1 enzyme thereby blocking androgen synthesis, with preferential lyase over hydroxylase inhibition, AR inhibition by antagonizing testosterone binding to the AR, which prevents binding of synthetic androgens to both mutant and wild-type AR, and finally, decreasing the amount of AR through degradation of the AR protein. These unique mechanisms of action are thought to be a possible promising agent for the treatment of CRPC (12). Thus, far, early Phase I testing (ARMOR 1) demonstrating a minimal side effect profile has led to a Phase II trial (ARMOR 2) evaluating efficacy by means of PSA response (NCT01709734). While TOK-001 and other promising drugs such as ARN-509 are in the pipeline (13), there remains a concern of whether the clinical trial endpoint that we are accustomed to that of overall survival is the most appropriate endpoint. To illustrate this point, results of a lyase inhibitor TAK-700 or orteronel have been presented in both the post-docetaxel and pre-docetaxel setting. While the eligibility criteria and study design appears comparable to contemporary clinical trials such as the COU-AA-301 and COU-AA-302 as well as AFFIRM and PREVAIL trials with abiraterone and enzalutamide, respectively, the TAK-700 trials ELM-PC5 and ELM-PC4 were disappointingly negative. This raises a concern since this drug, along with a multitude of other drugs, may not necessarily be clinically inferior, but increasingly difficult to prove superiority or even equivalency given the landscape of drugs currently approved that has been shown to improve overall survival.

Addressing the issue of resistance is of relevant importance given the notion that all CRPC tumors will fail treatment eventually. Provocative findings show gain-of-function mutation in 3β-hydroxysteroid dehydrogenase type 1 (3βHSD1) enzyme that renders an alternative pathway to resistance by inhibition of degradation and therefore provides a rapid route of conversion of an adrenal-derived dehydroepiandrosterone (DHEA) to the more potent androgen dihydrotestosterone (DHT), which can activate the AR (14). Increasing observation also abounds with regard to cross-resistance with novel androgen-signaling inhibitors where less than optimal responses are seen with enzalutamide post-abiraterone (15, 16) and vice-versa (response with abiraterone post-enzalutamide) (17). This phenomenon of cross-resistance may also be seen with the use of taxanes (18) with increasing recognition that taxanes inhibit AR translocation with resultant inferior responses to docetaxel after abiraterone initiation (19). The concurrent use of glucocorticoids has also been touted as a possible mechanism of resistance, especially in later stages of disease although this has been widely debated (20). Identification of AR splice variants as a mechanism of resistance may better predict the upfront resistance to the androgen-signaling inhibitors, which may guide therapy as recently shown in lack of response to either abiraterone or enzalutamide in tumors harboring the AR-V7 splice variant (21). In addition, better characterization of genetic and chromosomal rearrangements lends insights to potential targeting. For instance, one of the most prevalent oncogenes expressed in prostate cancer is that of ETS-related gene 1 (ERG) (22) and members of the ETS family of transcription factors (ERB, ETV1, ETV4) are placed under the control of an androgen-regulated promoter that occurs in the transmembrane protease, serine 2 (TMPRSS2), resulting in a gene fusion TMPRSS2-ERG that occurs in up to 50% of localized or metastatic prostate cancer. This may be relevant with addressing not only progression but also averting resistance or determining drug sequence patterns since response to abiraterone seem to be better in those who harbor the gene fusion (23, 24). Another interesting observation is the relationship between this gene fusion with PTEN. An invasive prostate cancer phenotype is promoted in phosphoinositide 3-kinase (PI3-kinase) activation (25) or PTEN loss (26). The cross-talk between PI3-kinase and androgen-signaling (27) offers a pharmacologic opportunity to achieve dual inhibition of compensatory pathways to avert the reciprocal feedback loop. Similar reciprocal relationship has been observed with androgen repression and c-met signaling with resultant epithelial–mesenchymal transition (EMT) and stem-cell like phenotype. In addition to the aforementioned genomic aberrations, deletion in NKX3.1, amplification in C-Myc, deletion of p53, deletion in CHD1, RB1, FOXA1, SPOP, and the HSP70/HSP90 chaperone complex have all been described (28, 29). Recently, a novel approach to targeting the bromodomain chromatin-binding protein BRD4, which interacts with the N-terminal domain of the AR using a small molecule JQ1, was reported (30). While similar to enzalutamide in its ability to inhibit AR recruitment, it improves upon enzalutamide’s action in its downstream AR binding, transcriptional regulation, and induction of TMPRSS2-ERG gene oncogenic function.

In summary, much progress has been made in the past decade alone in mCRPC therapy, but a lot remains to be done to further elucidate mechanisms of resistance and to circumvent these resistance mechanisms. The AR remains to be a versatile foe from progression of localized prostate cancer to advanced and metastatic prostate cancer and beyond castration-resistance. Provocative findings of transcriptional regulatory pathways and methods of inhibiting them will usher in an exciting era of targeting the AR in CRPC.

## Conflict of Interest Statement

The author declares that the research was conducted in the absence of any commercial or financial relationships that could be construed as a potential conflict of interest.
